# MiCroKiTS 4.0: a database of midbody, centrosome, kinetochore, telomere and spindle

**DOI:** 10.1093/nar/gku1125

**Published:** 2014-11-11

**Authors:** Zhengnan Huang, Lili Ma, Yongbo Wang, Zhicheng Pan, Jian Ren, Zexian Liu, Yu Xue

**Affiliations:** 1 Department of Biomedical Engineering, College of Life Science and Technology, Huazhong University of Science and Technology, Wuhan, Hubei 430074, China; 2 State Key Laboratory of Biocontrol, School of Life Sciences, Sun Yat-sen University, Guangzhou, Guangdong 510275, China

## Abstract

We reported an updated database of MiCroKiTS 4.0 (http://microkit.biocuckoo.org) for proteins temporally and spatially localized in distinct subcellular positions including midbody, centrosome, kinetochore, telomere and mitotic spindle during cell division/mitosis. The database was updated from our previously developed database of MiCroKit 3.0, which contained 1489 proteins mostly forming super-complexes at midbody, centrosome and kinetochore from seven eukaryotes. Since the telomere and spindle apparatus are critical for cell division, the proteins localized at the two positions were also integrated. From the scientific literature, we curated 1872 experimentally identified proteins which at least locate in one of the five positions from eight species. Then the ortholog detection was performed to identify potential MiCroKiTS proteins from 144 eukaryotic organisms, which contains 66, 45 and 33 species of animals, fungi and plants, respectively. In total, 87 983 unique proteins with corresponding localization information were integrated into the database. The primary references of experimentally identified localizations were provided and the fluorescence microscope figures for the localizations of human proteins were shown. The orthologous relations between predicted and experimental localizations were also present. Taken together, we anticipate the database can serve as a useful resource for further analyzing the molecular mechanisms during cell division.

## INTRODUCTION

**Figure 1.**
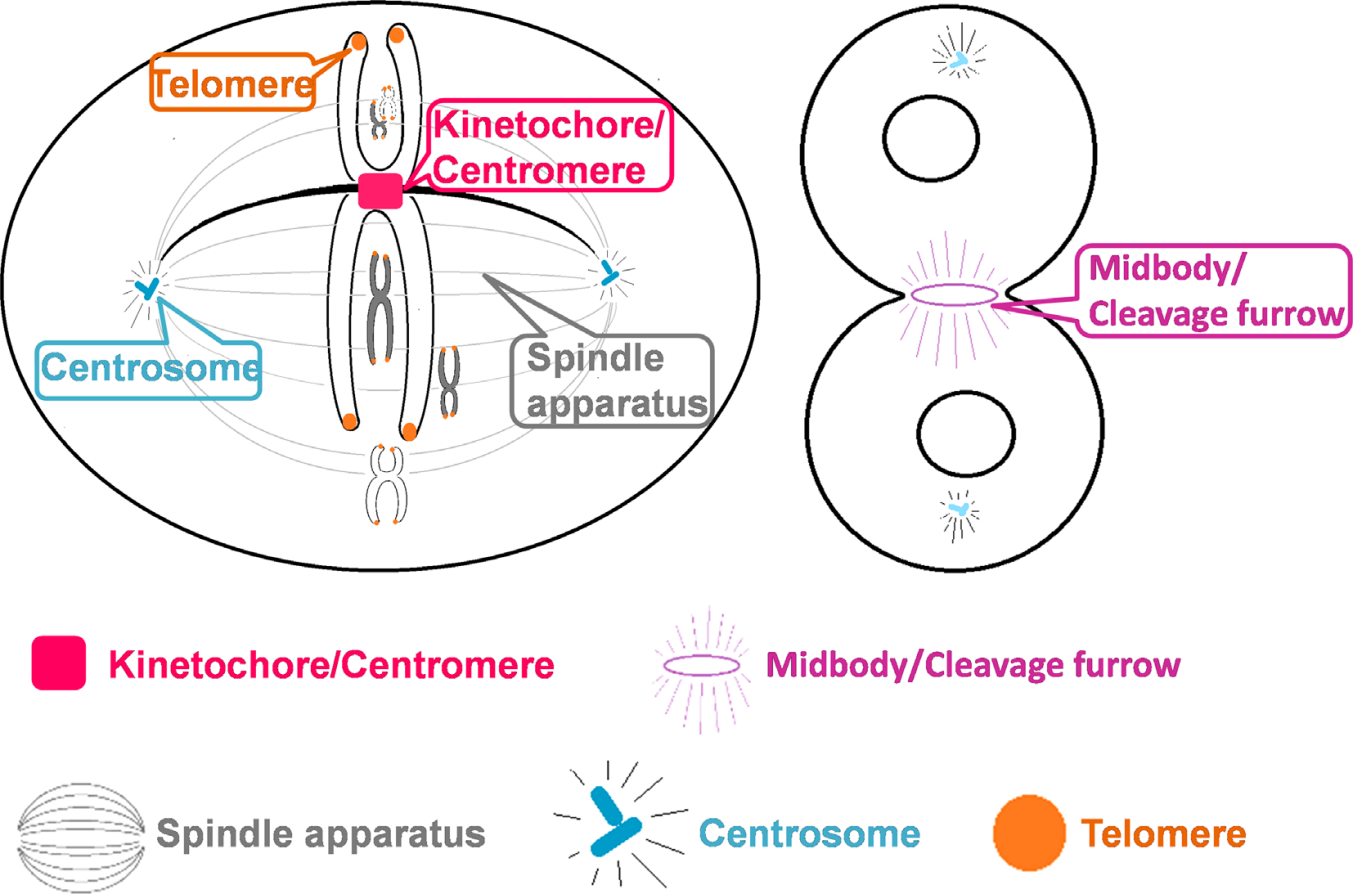
The schematic diagram for the five subcellular positions including midbody/cleavage furrow/bud neck/phragmoplast, centrosome/spindle pole body, kinetochore/centromere, telomere and spindle apparatus in eukaryotic cells.

In eukaryotic cells, a large number of proteins spatially and temporally localize at distinct subcellular positions and organize various super-complexes to orchestrate the chromosome segregation during cell division/mitosis (1). For example, the centrosome of animal cells, the spindle pole body in budding yeast and homologous structures in other species contain hundreds of proteins and act as the microtubule-organizing center (MTOC) (Figure 1) (2–5). Besides the nucleation and organization of microtubules and mitotic/meiotic spindles for attaching chromosomes during mitosis or meiosis, centrosome also plays critical roles in a variety of biological processes, such as primary cilia formation (4,5) and intracellular trafficking (4,5). The aberrance of centrosome or centrosomal proteins has been involved in the misregulation of cell cycle, genetic diseases (6) and cancers (7). For example, Lingle *et al.* found that the centrosomal amplification is highly associated with chromosomal instability (CIN) and may participate in breast tumor development and progression (8). Also, the tight interactions between microtubules and chromosomes are mediated by centromere via the attachment site kinetochore, which contains hundreds of proteins forming in super-complexes (Figure 1) (9). Centromere/kinetochore transmits the power from spindle microtubules for the chromosome movement (10), and serve as the checkpoint for cell division control to ensure all sister chromatids can be correctly and averagely delivered into daughter cells (11). The aberrance of centromere/kinetochore generates missegregation of chromosomes, CIN and anaphase lagging chromosomes (12,13), which are frequently observed in cancer cells (13,14). In addition, as the final stage of cell division, cytokinesis comprises a number of complicated processes including the average distribution of intracellular contents and the separation of two daughter cells (15–18). Numerous proteins are involved in cytokinesis through the cooperation in midbody/cleavage furrow, for which the conserved structures in yeast and plants are bud neck and phragmoplast, respectively (Figure 1) (18,19). Obviously, cytokinesis is critical for cell division, while the failure of this process might be involved in cancers (20). Taken together, a comprehensive identification of proteins located at centrosome, kinetochore and/or midbody is critical for further understanding the molecular mechanisms of cell division/mitosis.

Besides the distribution of mother cell contents equally into two daughter cells, the preservation of the chromosomal integrity and stability is also critical for cell cycle (21). As an intrinsic ‘mitotic clock’, telomere monitors the chromosome end-replication to ensure its length through the interactions of numerous proteins with telomeric DNA sequences (22,23). The aberrance of telomere is highly associated with various human diseases, such as ageing syndromes and cancers (24–26). For example, the shortened telomeres are associated with Werner Syndrome, a premature aging syndrome (27), whereas Chin *et al.* identified that transition through telomere crisis is crucial for the progression of breast cancers (28). A number of proteins located at midbody, centrosome or kinetochore can also translocate at telomere. For example, tankyrase, a human poly(ADP-ribose) polymerase, locates at centrosomes in mitosis, but colocalizes with a telomeric regulator TRF1 at telomeres during interphase (29). Also, two spindle assembly checkpoint proteins BubR1 and Mad2 can localize at kinetochore, but also colocalize with TRF1 at telomeres during mitosis, and form a link between the mitotic spindle and telomeres (30). Moreover, an E3 ubiquitin ligase Rnf8 localizes at midbody during cytokinesis (31), but can also translocate to uncapped telomeres for the chromosome end protection (32). Given the tight associations of telomere with midbody, centrosome and kinetochore, a systematic collection of telomeric proteins can provide helpful information for further studies on cell cycle and human health. In addition, a number of microtubule-associated proteins dispersedly localize at the spindle apparatus but not limited to centrosome or kinetochore. For example, a proteomic analysis together with further immunofluorescence assays identified at least six spindle proteins (33). Also, a Mad2 homolog, MAD2B, interacts and colocalizes with the clathrin light chain A at the mitotic spindle (34). Thus, the integration of mitotic/meiotic spindle proteins can also be helpful for further understanding the cell division.

With numerous experimental studies carried out to dissect the proteins localized in these subcellular positions, a handful of computational efforts have also been contributed. For example, the Cildb database was developed by Arnaiz *et al.* for centrosome and cilia proteins (35), while Nogales-Cadenas *et al.* constructed the CentrosomeDB database for human centrosomal proteins (36) and Alves-Cruzeiro *et al.* updated it to contain centrosomal proteins in *Drosophila melanogaster* (37). We also developed the MiCroKit database to maintain the proteins which were identified to localize in positions including centrosome, kinetochore and midbody for seven model organisms (38). Besides the database constructions, computational predictions and analyses were also performed. For example, Chen *et al.* developed the MicekiPred software to predict potential midbody, centrosome and kinetochore proteins (39), while recently Kuhn *et al.* and Azimzadeh *et al.* analyzed the evolutionary history of centrosome proteins (40,41). Furthermore, our computational studies showed that the positions including midbody, centrosome and kinetochore enriched KEN-box and D-box proteins (42), and the proteins regulated by Polo-like kinases (Plks) through phosphorylation and phospho-binding (43).

In this study, we greatly improved the MiCroKit 3.0 database through extending the types of localizations including spindle apparatus and telomere, and developed the MiCroKiTS (Midbody, Centrosome, Kinetochore, Telomere and Spindle) 4.0 database. From literature, we manually collected 1872 MiCroKiTS proteins among eight model organisms, which were two fungi including *Saccharomyces cerevisiae* and *Schizosaccharomyces pombe*, five animals including *Caenorhabditis elegans*, *D. melanogaster*, *Xenopus laevis*, *Mus musculus* and *Homo sapiens* and one plant *Arabidopsis thaliana*. Furthermore, based on the conception that orthologs among different organisms might share similar localizations in these subcellular positions, the orthologs for the experimentally identified MiCroKiTS proteins among 144 eukaryotes including 66 animals, 45 Fungi and 33 plants were detected. All the experimentally identified MiCroKiTS proteins and their orthologs were integrated into the MiCroKiTS 4.0 database, which contains 87 983 proteins in total. The source references, ortholog relationships and other annotations were provided for MiCroKiTS proteins in the database. Taken together, the MiCroKiTS 4.0 database could serve as a useful data resource for further studies of the molecular mechanisms for cell division.

## CONSTRUCTION AND CONTENT

In this study, we defined the MiCroKiTS proteins as the proteins which have localizations in any of the subcellular positions including centrosome/spindle pole body, kinetochore/centromere, mitotic/meiotic spindle, midbody/cleavage furrow and telomere. To construct a reliable data resource, we manually curated the experimentally identified MiCroKiTS proteins from literatures (published before 1 June 2014 in PubMed) in eight model organisms, which were two fungi including *S. cerevisiae* and *S. pombe*, five animals including *C. elegans*, *D. melanogaster*, *X. laevis*, *M. musculus* and *H. sapiens*, and one plant *A. thaliana*. With the rationale established previously (38), only the proteins which were unambiguously observed to be localized at these super-complexes under fluorescent microscope were collected.

To collect the MiCroKiTS proteins, a number of keywords were employed to search the literature in PubMed. For centrosome/spindle pole body, kinetochore/centromere, midbody/cleavage furrow, the keywords were adopted as previously described (38), while additional keywords were considered for plants. For example, the terms ‘MTOC’ and ‘phragmoplast’ were used to search similar structures for centrosome and midbody in plants, respectively. For spindle apparatus and telomere, the keywords ‘spindle’ and ‘telomere’ were employed. To simplify the descriptions in this study, the terms ‘centrosome’, ‘kinetochore’, ‘midbody’, ‘spindle’ and ‘telomere’ were used to representing these super-complexes and similar structures. In total, we collected 1872 MiCroKiTS proteins, which contain 2277 experimentally identified localizations. In comparison with MiCroKit 3.0 database, 383 new proteins and 508 newly reported localizations of both previously collected and new proteins were added. Furthermore, to provide an intuitive presentation for MiCroKiTS localizations, the first published fluorescence evidence for localizations of human MiCroKiTS proteins were obtained from the literature.

To provide information for species beyond the eight model organisms, homologous detections were performed to search orthologs, which might be potential MiCroKiTS proteins and could be helpful for further studies of these super-complexes. The reference proteomes from 143 genome-sequenced eukaryotes including 65 animals, 45 fungi and 33 plants were downloaded from Ensembl database (44), while the reference proteome of *X. laevis* was unavailable. As previously described (38,45,46), the strategy of reciprocal best hits (47) with Basic Local Alignment Search Tool (BLAST) package (48) were employed to detect orthologs of MiCroKiTS proteins from the eight model organisms in other species. Based on the concept that orthologs might have similar localizations in these subcellular positions, the localizations of orthologs were predicted as the homologous experimentally identified MiCroKiTS proteins. In total, 86 111 orthologs were predicted as potential MiCroKiTS proteins, which were also integrated into the MiCroKiTS 4.0 database. The numbers of proteins with different localizations among different organisms were summarized in Supplementary Table S1. All the proteins in the database were annotated with source references and other annotations from UniProt database (49) to provide brief introductions. All localizations and sequences of proteins in MiCroKiTS were available for download at http://microkit.biocuckoo.org/download.php.

## USAGE

**Figure 2.**
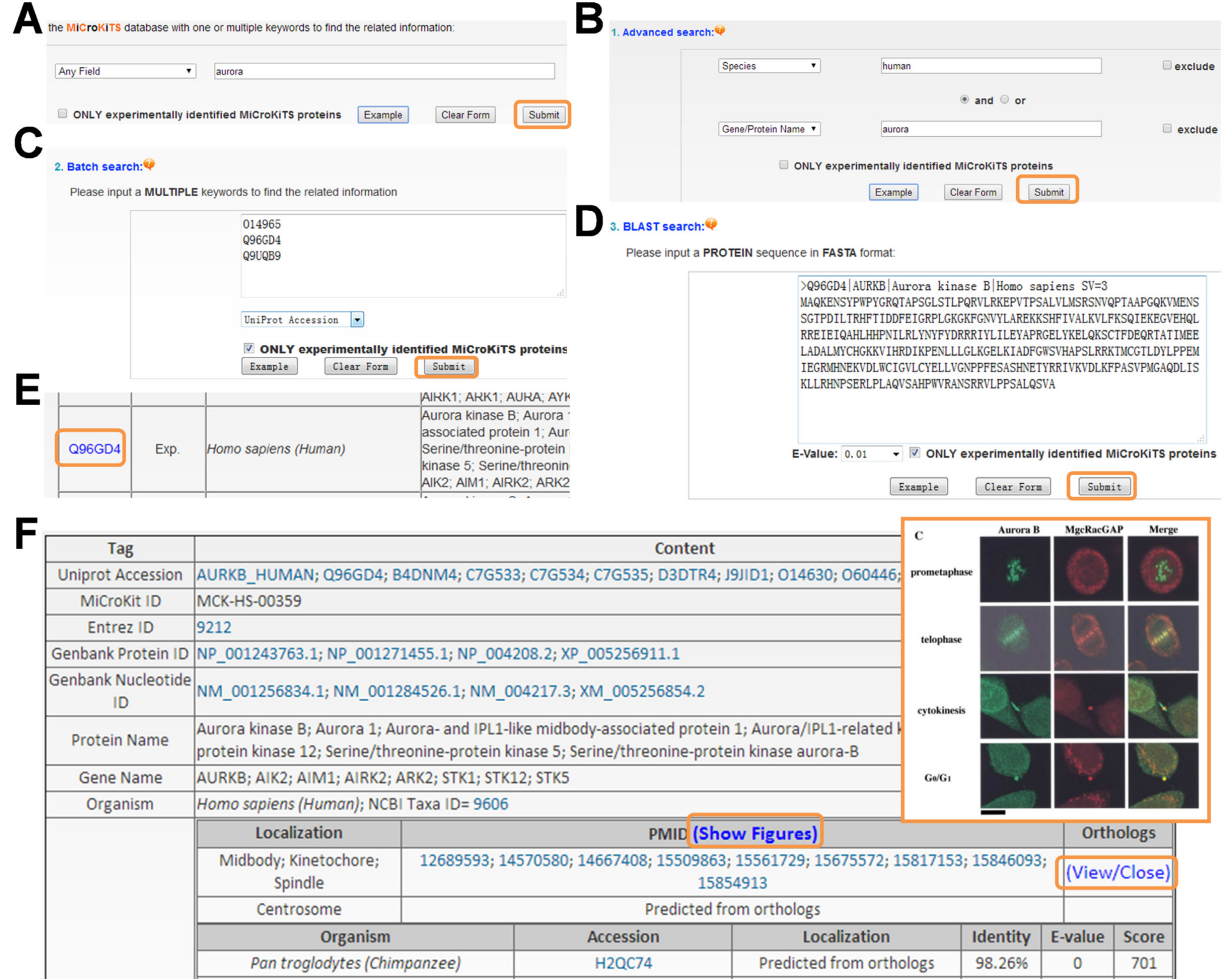
The search options of MiCroKiTS database. (A) The database can be directly queried with one or multiple keywords. (B) The ‘Advanced search’ option allows users to submit a combination of two terms for searching. (C) The database could be searched to retrieve a list of proteins through submitting a list of keywords such as UniProt accessions. (D) The database can be queried with a protein sequence in FASTA format to find identical or homologous proteins. (E) The protein list derived from the search options. (F) The details of the MiCroKiTS protein Aurora kinase B in *H. sapiens*, while the fluorescence microscope figure for the localizations can also be visualized.

To provide convenient usage, the MiCroKiTS 4.0 database web interface was designed in a user-friendly manner for search and browse. The website contains four search options including one/multiple keywords-based simple search (Figure 2A), ‘Advanced search’ based on a combination of multiple keywords (Figure 2B), multiple keywords based ‘Batch search’ (Figure 2C) and protein sequence-based ‘BLAST search’ (Figure 2D). For example, if a keyword ‘aurora’ in ‘Any Field’ was submitted for a simple search (Figure 2A), the website will return a list of MiCroKiTS proteins, such as Aurora kinase B from *H. sapiens* in a tabular format with accession, species, and protein/gene names/aliases (Figure 2E). By clicking the accession ‘Q96GD4’, user could visit the webpage of human Aurora kinase with detailed annotation including localizations, PubMed IDs of source references and the orthologs (Figure 2F). For human MiCroKiTS proteins, the representing fluorescent microscope figures were provided by clicking the ‘Show Figures’ (Figure 2F). Furthermore, two terms specified in two areas and combined with operators of ‘and’, ‘or’ and ‘exclude’ could be employed to perform a complex query in ‘Advanced Search’ (Figure 2B). For example, querying the database with ‘human’ in ‘Species’ and ‘aurora’ in ‘Gene/Protein Name’ will return three human aurora kinases (Figure 2B). Alternatively, user could submit a list of keywords to perform a batch search. For example, three human aurora kinases could be retrieved by submitting the list of their UniProt accessions (Figure 2C). Furthermore, user could submit a protein sequence in FASTA format in ‘BLAST Search’ to find homologous MiCroKiTS proteins (Figure 2D). For example, the sequence of human Aurora kinase B could be input in the FASTA format to search homologous proteins in the database. The ‘Advanced Search’, ‘Batch search’ and ‘BLAST Search’ will return the list of searching hits in a tabular format as the simple search (Figure 2E). In addition, there is a checkbox of ‘ONLY experimentally identified MiCroKiTS proteins’ for each search option (Figure 2). If the checkbox is selected, only experimentally identified MiCroKiTS proteins will be queried.

**Figure 3.**
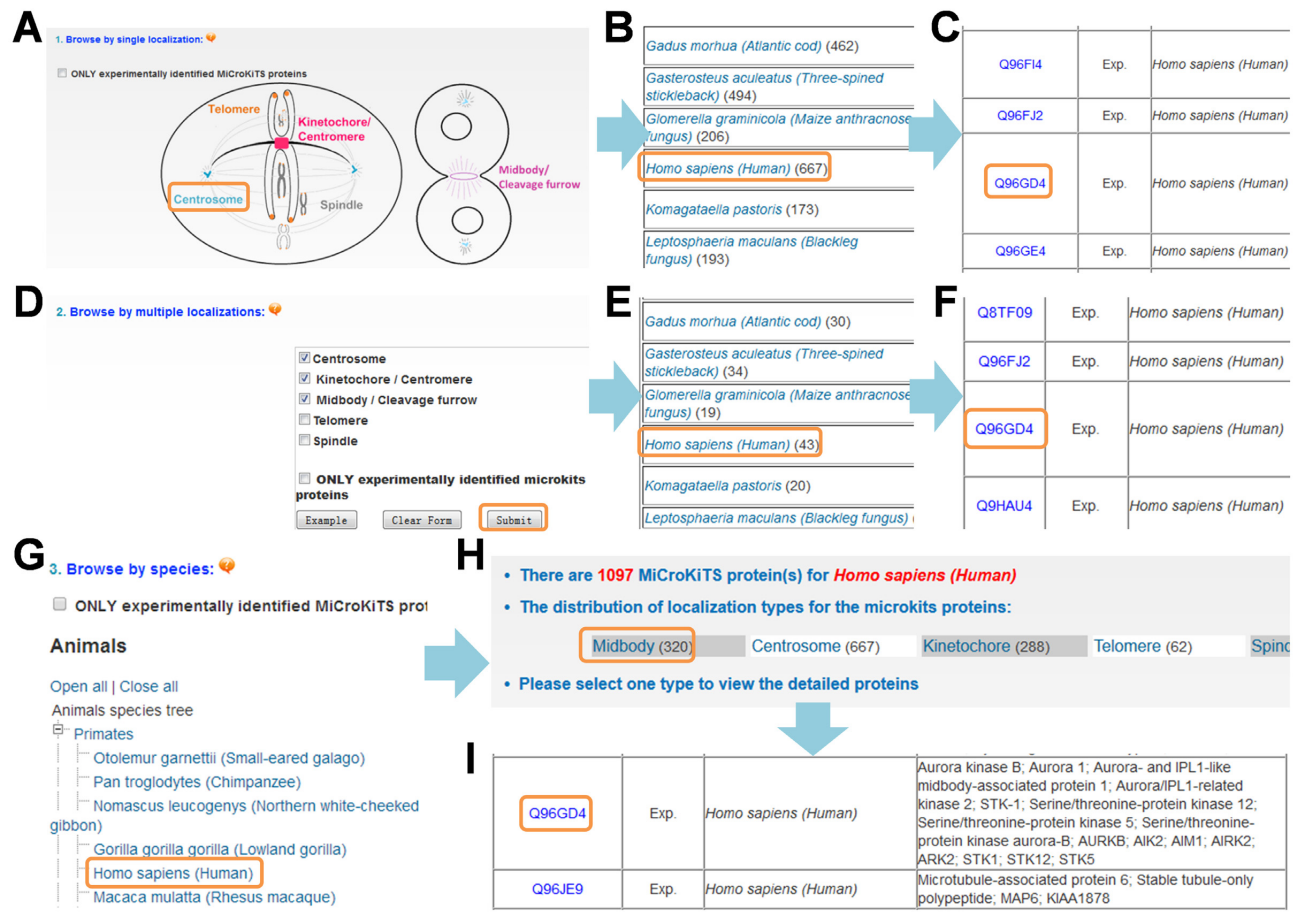
The MiCroKiTS database can be browsed by either localizations or by species. (A) Browse by single localization. (B) The distribution of centrosomal proteins in different organisms. (C) The list of centrosomal proteins in *H. sapiens.* (D) The proteins with multiple localizations can also be browsed. (E) For example, the distribution of proteins located at centrosome, kinetochore and midbody among different organisms can be shown. (F) The list of proteins located in all of the three positions in *H. sapiens*. (G) Browse by species. (H) The distribution of human MiCroKiTS proteins in different subcellular positions. (I) The list of midbody proteins in *H. sapiens*.

For convenient browse in MiCroKiTS database, we developed three options including single localization-based browse, multiple localizations-based browse and browse by species. For example, through clicking the ‘Centrosome’ in the single localization browse option (Figure 3A), the distribution of centrosome proteins among organisms was returned (Figure 3B), while centrosomal proteins, such as Aurora kinase B from *H. sapiens* were listed after further clicking the species name ‘*Homo sapiens (Human)*’ (Figure 3C). Furthermore, the multiple localizations-based browse option enable users to find the proteins localized in all the selected subcellular positions (Figure 3D). For example, if the checkboxes of centrosome, kinetochore and midbody were selected (Figure 3D), the MiCroKiTS proteins localized in all the three subcellular positions among different organisms were shown (Figure 3E). These proteins could be listed through clicking the species name (Figure 3F). Alternatively, the MiCroKiTS database could be browsed by organisms. For example, after clicking the ‘*Homo sapiens (Human)*’ in the list (Figure 3G), the distribution of human MiCroKiTS proteins in the subcellular regions was shown (Figure 3H), while the human midbody proteins, such as Aurora kinase B, could be listed through clicking ‘Midbody’ (Figure 3I). Again, the checkboxes of ‘ONLY experimentally identified MiCroKiTS proteins’ were provided for exclusively browsing the experimentally identified MiCroKiTS proteins (Figure 2A, D and G).

## DISCUSSION

During cell division, a large number of proteins are transiently recruited to distinct subcellular positions, such as midbody/cleavage furrow, centrosome/spindle pole body, kinetochore/centromere, mitotic/meiotic spindle and telomere, and assemble various protein super-complexes for orchestrating the segregation of cell contents into daughter cells (2–5,9–11,15–18,50–58). These proteins play critical roles in various cellular processes, while their aberrances were heavily related with diseases and cancers (6,7,12,20,24–26,53). Thus, systematic dissecting the proteins localized in these positions will be helpful for further understanding their functional roles and regulatory mechanisms.

**Figure 4.**
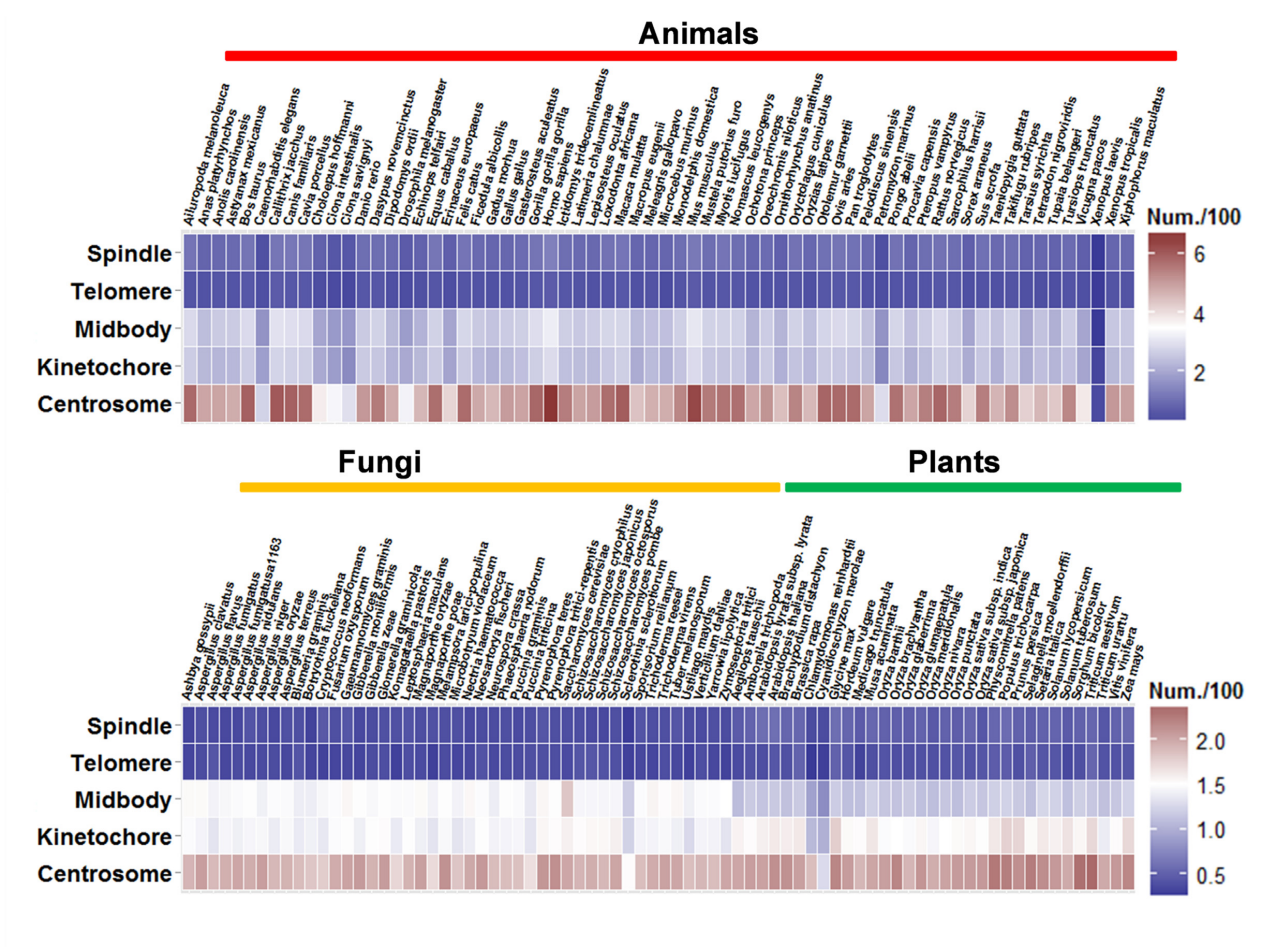
The heatmap about the distribution of MiCroKiTS proteins in the MiCroKiTS 4.0 database for different localizations among 144 eukaryotic species from different kingdoms. The upper part was the results for the 66 animals, and the lower section was the results for the 33 plants and 45 fungi.

In this study, we updated the database of MiCroKit 3.0 into MiCroKiTS 4.0 for more organisms and more types of subcellular positions including spindle apparatus and telomere. In total, 1872 experimentally identified MiCroKiTS proteins with 2277 localizations were collected in eight model organisms. Furthermore, homologous detections were performed to find orthologs in species beyond the eight model organisms for experimentally identified MiCroKiTS proteins to search potential MiCroKiTS proteins, which were also integrated into the database. The distribution of proteins in centrosome, kinetochore, spindle apparatus, midbody and telomere were summarized and presented in Figure 4. Because the reference proteome for *X. laevis* was unavailable, only known MiCroKiTS proteins were collected (Figure 4). From the result, it was observed that the centrosome has most proteins, while there were more proteins localized in kinetochore and midbody than spindle apparatus and telomere (Figure 4). Also, the numbers of MiCroKiTS proteins per localization vary greatly among different kingdoms, but are similar in the same kingdom (Figure 4). However, further experimental studies are still needed to verify the observations, while orthologs among distantly related species, such as organisms in different kingdoms, should be carefully considered. Taken together, here we updated the MiCroKit 3.0 database, which only contains proteins for three super-complexes in seven organisms, to MiCroKiTS 4.0 database for subcellular positions in 144 species. We believed that the update will make the database more helpful for the further computational or experimental studies. The MiCroKiTS database will be routinely updated to maintain more information for systematic understanding of the molecular mechanisms and function roles of the MiCroKiTS proteins during cell cycle.

## SUPPLEMENTARY DATA

Supplementary Data are available at NAR Online.
